# Determinants of inequalities in the quality of Brazilian diet: trends in 12-year population-based study (2003–2015)

**DOI:** 10.1186/s12939-018-0784-2

**Published:** 2018-06-07

**Authors:** Aline Veroneze de Mello, Flávia Mori Sarti, Jaqueline Lopes Pereira, Moisés Goldbaum, Chester Luiz Galvão Cesar, Maria Cecilia Goi Porto Alves, Regina Mara Fisberg

**Affiliations:** 1 Department of Nutrition, School of Public Health, University of São Paulo, São Paulo, Brazil; 2 School of Arts, Sciences and Humanities, University of São Paulo, São Paulo, Brazil; 3 Department of Nutrition, School of Public Health, University of São Paulo, São Paulo, Brazil; 4 Department of Preventive Medicine, School of Medicine, University of São Paulo, São Paulo, Brazil; 5 Department of Epidemiology, School of Public Health, University of São Paulo, São Paulo, Brazil; 6 Health Institute of São Paulo, São Paulo, Brazil; 7 Department of Nutrition, School of Public Health, University of São Paulo, Avenida Dr. Arnaldo, 715 – Cerqueira Cesar, São Paulo, SP 01246-904 Brazil

**Keywords:** Diet, Food consumption, Diet quality index, Inequality in health, Income

## Abstract

**Background:**

Recent studies have explored the influence of socioeconomic inequalities on the diet quality. However, there is lack of evidence regarding the level of inequalities in dietary quality and its main contributing factors from population-based follow-up studies. The primary objective of this study was to investigate the level and the determinants of inequalities in diet quality in a representative sample of adolescents, adults and older adults resident in São Paulo, Brazil.

**Methods:**

Data from the Health Survey of São Paulo (ISA-Capital) were analyzed for 2003 (*n* = 2398), 2008 (*n* = 1662) and 2015 (*n* = 1742) surveys. Information on food consumption was obtained through 24-h dietary recall, and diet quality was assessed based on the Revised Brazilian Healthy Eating Index (BHEI-R). The descriptive variables were compared using 95% confidence interval. The scores of BHEI-R and its components were compared across age groups and year. The association between socioeconomic inequalities and diet quality was based on the estimation of concentration index.

**Results:**

We observed that the BHEI-R scores gradually improved over 12-years, with older adults showing the greatest improvement. The increase in overall population score was observed for total fruits, whole fruits, whole grains, oils and sodium. The main contributor to socioeconomic inequality in diet quality in 2003 was ethnic group, and in 2008 and 2015, it was per capita household income; age was a persistent factor of inequality in the population over the years. Concentration indices indicated that lower income individuals had higher BHEI-R scores in 2003; however, there was a shift in favor of higher income individuals in 2008 and 2015.

**Conclusions:**

Changes in the patterns of determination of inequalities according to age, ethnic group or income during the period analyzed show the existence of ongoing process of contribution of demographic and socioeconomic factors in the diet quality of individuals in a large urban center.

## Background

Diet quality is a modifiable risk factor that influences the development of chronic diseases such as obesity, type 2 diabetes mellitus, and cardiovascular diseases [1, 2]. It contributes to inequalities in health and, at the same time, is influenced by socioeconomic inequalities, such as access to education and health services, which in turn are associated with living conditions, such as income, occupation and educational attainment [1, 3–5].

The investigation of socioeconomic and demographic factors related to diet quality in the context of socioeconomic determinants of health has been explored in recent studies [6–9]. However, there is still no evidence regarding the level of inequality in the diet quality in population-based follow-up studies, neither is there analysis of the factors contributing to the inequalities.

Studies that assess factors associated with inequalities of health status or access to and use of health services have used the research methodology of the determinants of income inequalities [10–12]. Therefore, the present study proposes and aim to employ such methodology to investigate level and the determinants of inequalities in diet quality and their magnitude in a representative sample of residents in São Paulo city, Brazil, in 2003, 2008 and 2015. This makes it the first Brazilian study to investigate the evolution and the determinants of inequalities in diet quality at the population level.

## Methods

Data from three cross-sectional samples of individuals from São Paulo city, interviewed in the Health Survey of São Paulo (ISA-Capital) conducted in 2003, 2008 and 2015, was used to perform the analysis. ISA-Capital is an observational study with a probabilistic sample of individuals living in the urban area of São Paulo (SP). São Paulo is the largest city in South America, representing roughly 6% of Brazilian population and 10% of Brazilian Gross Domestic Product (GDP), according to the Brazilian Institute of Geography and Statistics (IBGE). São Paulo’s economy is predominantly based on commercial and financial activities, being the city with the 10th largest GDP in the world.

**Fig. 1:**
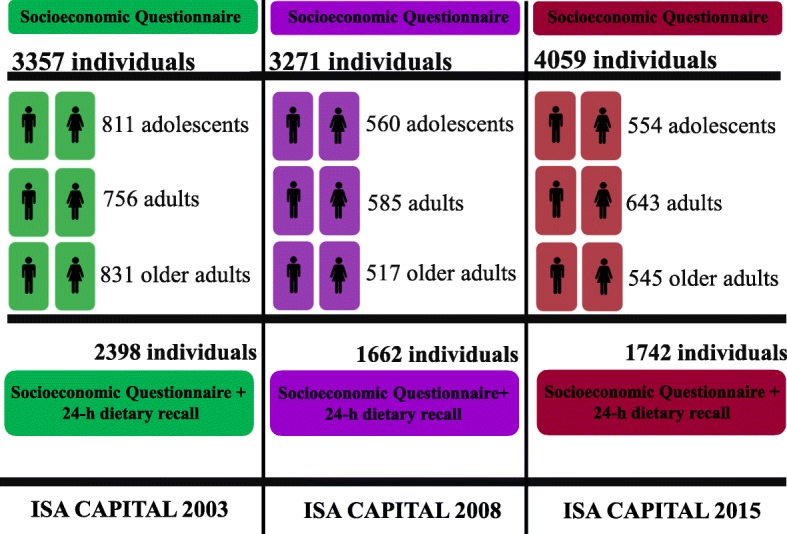
Design of the Health Survey of São Paulo, according to period of the survey and sample details. ISA 2003, 2008, 2015 (SP). Brazil. 2003–2015

The selection process was based in two-stage cluster sampling stratified by conglomerates (urban census tracts and households), in order to ensure representativeness at population level. Details of the 2003, 2008 and 2015 surveys, including the sampling design and the sample size calculation, were previously published [13–15]. The samples of the present study included participants residing in São Paulo aged 12 years or older, of both sexes, in the three years of the study (Fig. 1).

The study considered the definitions of World Health Organization (WHO) and Brazilian Statute for Children and Adolescents to categorize the age range due to constraints imposed within Brazilian laws; thus, adolescents were considered individuals aged between 12 and 19; adults were considered individuals aged between 20 and 59; and elderly were considered individuals aged over 60 years.

The information was collected at the participants households by trained interviewers, using a socioeconomic questionnaire structured in thematic blocks. Food consumption information was obtained from a 24-h dietary recall (24HR), during a face-to-face interview, following the procedures of the Multiple Pass Method (MPM), in which five sequential steps are applied: quick list, forgotten foods list, time and occasion, detail and review, and final probe of the recall, in order to increase the response rate and maintain the attention of the respondent [16].

Food consumption data were entered into the Nutrition Data System for Research 2014 (NDSR) software, following the standardization of food quantities in grams or milliliters [17, 18]. All 24HRs whose energy consumption were lower than 800 kcal or higher than 4000 kcal were verified to guarantee quality of the information.

Diet quality was evaluated based on calculation of the Revised Brazilian Healthy Eating Index (BHEI-R) [19], which includes 12 components: nine food groups (total fruit, whole fruit, total vegetables, dark green and orange vegetables and legumes, whole grains, milk and dairy products, meats, eggs and legumes, oils), two nutrients (saturated fat, sodium) and a component related to the consumption of total calories from solid fat, alcohol and added sugar (SoFAAS). The final BHEI-R score ranged from 0 to 100, with 0 being a very poor diet and 100 being an excellent diet.

Variables selected for the analysis of determinants of inequalities in diet quality included: age (12 to 19 years, 20 to 59 years or 60 years or more); sex (male or female); ethnic group (white or non-white: black, grayish-brown, yellow and indigenous); individual education (less than or equal to 4 years, 5 to 9 years, 10 to 12 years or 12 years or more); occupation (employed or unemployed, being included in the second category those effectively unemployed, and also students, housewives and retired individuals); household income per capita in adult equivalents (equals to the total household income, divided by the number of adult-equivalent individuals in the family: less than or equal to 1 minimum wage, or greater than 1 minimum wage); type of residence (house, apartment, shed or house of rooms); marital status (with or without partner); health insurance (yes or no); chronic diseases (presence or absence); physical activity level (minutes per day, according to WHO global recommendation) [20]; smoking habits (non-smoker, former smoker or current smoker); alcohol consumption (yes or no) and components of BHEI-R (being statistically significant in determination of socioeconomic inequalities: sodium and whole grains).

Data analysis was carried out on the Stata Data Analysis and Statistical Software (version 13) in survey mode, considering the complex sample design for selection of the strata (urban census tracts) at geographical level, followed by selection of household (primary sampling unit), using sampling weights for expansion of the representativeness at populational level in São Paulo city.

Unregistered variables were encoded as missings. Qualitative variables were described as frequency, percentage and 95% confidence interval and quantitative variables as average and standard error. Test of adherence to the normal distribution curve (Shapiro-Wilk) for BHEI-R and its respective components was performed. As the variables did not present normal distribution, the non-parametric Kruskal-Wallis test was applied, complemented by the nptrend test, considering the descriptive level of *p* < 0.05.

The estimation of household income per capita in adult equivalents, used for fractional classification of the individuals of the sample according to socioeconomic status in the population, was derived from the following equation:

1 $$ {\mathrm{e}}_{\mathrm{h}}={\left({\mathrm{A}}_{\mathrm{h}}+{\upphi \mathrm{K}}_{\mathrm{h}}\right)}^{\uptheta} $$

With: Ah = number of adult individuals (older than 14 years of age) in the household h; and Kh = number of individuals aged less than 14 years at home h. The parameters Φ and θ are set at 0.75, according to Deaton’s original weight proposal (1997) [21], in order to avoid underestimation of income effects based on the adoption of household income per capita, due to the lower requirements attributable to younger individuals in the domestic unit.

The analysis of inequality in diet quality among individuals in the population was based on the estimation of the concentration index (CI), detailed in previous study [22], using the following equation:

2 $$ \mathrm{CI}=\frac{2\ }{\mathrm{n}\upmu}\ {\sum}_{\mathrm{i}=1}^{\mathrm{N}}{\mathrm{y}}_{\mathrm{i}}\ {\mathrm{R}}_{\mathrm{i}}-1 $$

With: μ = mean of y (dependent variable: BHEI-R); Ri = fractional classification of the i-th individual in the income distribution (accumulated income of the i-th individual, in a population with N individuals); N = sample size.

The CI represents an inequality index which allows identification of income-related inequality in the diet quality of individuals. The analysis focuses on quality of food consumption among individuals with diverse economic status in Brazil, using the BHEI-R as measure of diet quality.

In order to identify the main determinants of diet inequality, the estimation of multiple linear regressions with dependent variable based on the concentration index of dietary quality was performed against independent variables representing individual characteristics (sex, age, ethnic group, physical activity, alcohol consumption, smoking habits, diet quality components sodium and whole grains, and chronic disease) and external factors (per capita household income, education, occupation, marital status, type of residence and health insurance), at a descriptive level of *p* < 0.05, using the following equation:

3 $$ {\mathrm{y}}^{\ast }={\upbeta}_1^{\hbox{'}}\cdot \mathrm{x}+{\upbeta}_2^{\hbox{'}}\cdot \mathrm{w}+\upvarepsilon $$

Where y is a vector of CI in diet quality, matrix x includes a set of variables of individual characteristics and matrix w includes other independent variables associated with external factors. The dependent variable (y) describes differences in diet quality according to socioeconomic status of the individuals, and the coefficients (βk) of the multiple linear regression models are estimated against individual’s characteristics (x) and other factors that determine the diet quality (w). Matrix x includes gender, age group, ethnic group, health behaviors, and educational attainment to measure the influence of personal features in diet quality; whilst matrix w includes external factors affecting diet quality: household income per capita in adult equivalents, occupation status, marital status, and type of residence. Variables included in the regressions were tested for multicollinearity and all the variance inflation factors (VIF) values were lower than 10, indicating that there is not multicollinearity.

Thus, the analysis pursued the identification of patterns in evolution and determinants in CI which was calculated to represent an index measuring inequality in diet quality (represented by BHEI-R) according to the socioeconomic position of the individuals (represented by household income per capita in adult equivalents) in comparison to the total population.

The concentration index and the regression models were estimated with and without adolescents, in order to verify potential problems regarding the inclusion of variables referring to educational attainment, occupation, marital status, alcohol consumption and smoking habit; nevertheless, results obtained in the analysis of adult population were very similar in relation to evolution and determinants of diet quality of the whole population in the study.

The individuals interviewed in the three surveys were informed about the purposes of the research, and were asked to sign a consent form to voluntarily accept to participate in the research. Both the original research projects of the ISA and the present study were approved by the Ethics Committee of the School of Public Health at the University of São Paulo.

## Results

There were significant changes in the population of the São Paulo city during the 12-year period encompassed by the surveys (Table 1). In 2008, the proportion of individuals with education of 10 to 12 years, with income less than/equal to a minimum wage, living at home and with presence of chronic disease was higher than in 2003. In 2015, compared to 2003, there was a higher proportion of older adults, of people without partners, unemployed, with education from 5 to 9 years, living in a house of rooms, not consuming alcohol and with chronic disease.

**Table 1:** Characteristics of the population*, according period of the survey. ISA 2003, 2008, 2015. (SP), Brazil, 2003–2015

Variables*	ISA 2003		ISA 2008		ISA 2015	
	n**	% (CI 95%)***	n**	% (CI 95%)***	n**	% (CI 95%)***
Sex						
Male	1186	46.06 (43.38–48.77)	722	46.90 (44.37–49.44)	837	50.02 (47.17–52.86)
Female	1212	53.94 (51.23–56.62)	940	53.10 (50.56–55.64)	905	49.98 (47.14–52.83)
Age group (years)						
12–19	811	18.93 (16.98–21.05)	560	15.14 (12.93–17.34)	554	23.17 (20.87–25.66)
20–59	756	68.18 (66.01–70.27)	585	71.00 (68.00–74.01)	643	54.15 (51.45–56.82)
≥ 60	831	12.89 (11.30–14.66)	517	13.86 (11.62–16.10)	545	22.68 (19.90–25.71)
Ethnic group						
White	1517	65.51 (61.63–69.20)	963	58.46 (52.72–64.20)	840	49.59 (45.76–53.41)
Non-white	870	34.49 (30.80–38.37)	697	41.54 (35.80–47.28)	888	50.41 (46.59–54.24)
Education						
≤ 4 years	1048	35.60 (31.88–39.53)	595	22.65 (19.05–26.70)	265	12.33 (10.50–14.44)
5–9 years	518	22.96 (20.20–25.97)	450	24.16 (19.11–30.04)	607	30.22 (27.69–32.88)
10–12 years	454	21.01 (18.21–24.11)	375	33.39 (28.08–39.15)	573	33.95 (31.31–36.67)
≥ 12 years or more	347	20.43 (16.41–25.13)	216	19.80 (14.59–26.31)	292	23.50 (20.16–27.21)
Household income per capita						
< 1 MW	1123	42.06 (37.75–46.49)	1263	74.88 (69.07–79.91)	769	50.07 (45.13–55.00)
≥ 1 MW	1275	57.94 (53.51–62.25)	399	25.12 (20.09–30.93)	640	49.93 (45.00–54.88)
Occupation						
Unemployed	842	25.54 (22.45–28.88)	603	25.74 (22.63–29.12)	955	46.22 (43.42–49.05)
Employed	1531	74.46 (71.12–77.55)	1028	74.26 (70.88–77.37)	761	53.78 (50.95–56.58)
Marital status						
With partner	1661	71.38 (67.99–74.54)	1101	70.13 (65.64–74.27)	1068	56.07 (53.20–58.90)
Without partner	713	28.62 (25.46–32.01)	536	29.87 (25.73–34.36)	666	43.93 (41.10–46.80)
Type of residence						
House	2043	81.10 (72.78–87.31)	1533	93.04 (88.26–95.96)	1339	77.17 (70.82–82.49)
Apartment	305	17.75 (11.66–26.09)	116	6.30 (3.49–11.12)	197	13.65 (9.56–19.14)
Shed	6	0.33 (0.09–1.13)	9	0.53 (0.12–2.27)	2	0.13 (0.03–0.57)
House of rooms	17	0.82 (0.16–4.25)	3	0.13 (0.03–0.75)	149	9.04 (5.79–13.85)
Physical activity						
Active	613	21.93 (18.99–25.18)	366	18.11 (15.00–22.69)	442	22.93 (20.36–25.71)
Inactive	1759	78.07 (74.82–81.01)	1296	81.89 (78.31–84.99)	1277	77.07 (74.29–79.64)
Smoking habits						
Non-smoker	1651	66.38 (63.48–69.15)	1155	64.17 (60.45–67.87)	1261	71.23 (68.38–73.92)
Former smoker	371	14.72 (12.40–17.40)	274	16.04 (13.21–18.86)	261	14.79 (12.57–17.31)
Current smoker	360	18.90 (16.53–21.52)	232	19.79 (16.44–23.14)	214	13.98 (12.19–15.99)
Alcohol consumption						
No	1350	48.49 (44.79–52.20)	962	47.89 (44.69–51.08)	1292	69.35 (65.69–72.79)
Yes	1026	51.51 (47.80–55.21)	699	52.11 (48.92–55.31)	442	30.65 (27.21–34.31)
Chronic disease						
No	1834	84.56 (82.33–86.55)	1222	79.46 (76.74–81.94)	1268	76.13 (73.75–78.35)
Yes	533	15.44 (13.45–17.67)	439	20.54 (18.06–23.26)	464	23.87 (21.65–26.25)
Health Insurance						
No	1584	63.07 (58.80–67.16)	1126	64.60 (59.07–69.76)	1108	61.00 (56.79–65.05)
Yes	772	36.93 (32.84–41.20)	536	35.40 (30.24–40.93)	622	39.00 (34.95–43.21)

*Analyzes considered complex sample design for representativeness of the population of São Paulo

**Individuals interviewed in the sample. Exclusion of individuals with missing information

***CI 95% = Confidence Interval 95%. Confidence intervals do not overlap, i.e., there is (at least) 95% confidence that values are not equal in the population

MW = minimum wage

There was a gradual improvement in diet quality of São Paulo residents in 12 years, with the BHEI-R score departing from 54.70 (Standard Error, SE = 0.51) in 2003 and reaching 58.38 (SE = 0.39) in 2015 (*p* < 0.001). Regarding dietary quality of different age groups, there was a deterioration of the diet quality of adolescents between 2003 and 2008 (from 50.76, SE = 0.57 to 50.33, SE = 0.52), followed by improvement in 2015 (54.46, SE = 0.56). There was a gradual improvement in diet quality of adults and older adults, and the older had better overall scores in all periods (Table 2).

**Table 2:** BHEI-R and its components, according age groups. ISA 2003, 2008, 2015 (SP), Brazil, 2003–2015

BHEI-R Components* (maximum score)	Total				Adolescents				Adults				Older Adults			
	2003 (n = 2398)	2008 (n = 1662)	2015 (n = 1742)	p**	2003 (*n* = 811)	2008 (*n* = 560)	2015 (*n* = 554)	p**	2003 (*n* = 756)	2008 (*n* = 585)	2015 (*n* = 643)	p**	2003 (*n* = 831)	2008 (*n* = 517)	2015 (*n* = 545)	p**
	Mean (SE)	Mean (SE)	Mean (SE)		Mean (SE)	Mean (SE)	Mean (SE)		Mean (SE)	Mean (SE)	Mean (SE)		Mean (SE)	Mean (SE)	Mean (SE)	
Total fruit (5)	1.83 (0.07)	1.94 (0.08)	2.46 (0.07)	**< 0.001** **< 0.001**	1.64 (0.10)	1.36 (0.08)	1.85 (0.12)	0.059 **0.038**	1.72 (0.09)	1.88 (0.11)	2.39 (0.10)	**< 0.001** **< 0.001**	2.71 (0.10)	2.88 (0.14)	3.23 (0.11)	**0.001** **< 0.001**
Whole fruit (5)	1.54 (0.08)	1.92 (0.09)	2.32 (0.08)	**< 0.001** **< 0.001**	1.24 (0.09)	1.22 (0.08)	1.54 (0.11)	**0.010** **0.005**	1.41 (0.09)	1.86 (0.12)	2.21 (0.10)	**< 0.001** **< 0.001**	2.67 (0.11)	3.01 (0.14)	3.38 (0.12)	**< 0.001** **< 0.001**
Total vegetable (5)	4.12 (0.05)	4.17 (0.06)	4.05 (0.05)	0.132 0.157	3.72 (0.08)	3.59 (0.12)	3.74 (0.10)	0.801 0.526	4.20 (0.07)	4.25 (0.07)	4.09 (0.06)	0.117 0.058	4.32 (0.05)	4.34 (0.07)	4.27 (0.09)	0.460 0.801
DGOV&L (5)^a^	3.46 (0.08)	3.61 (0.09)	3.30 (0.07)	**0.047** 0.081	2.95 (0.09)	2.86 (0.11)	3.05 (0.13)	0.401 0.918	3.57 (0.11)	3.73 (0.11)	3.32 (0.11)	**0.036** **0.010**	3.64 (0.07)	3.79 (0.11)	3.51 (0.11)	0.239 0.321
Total grains (5)	4.59 (0.03)	4.52 (0.04)	4.54 (0.03)	**0.013** **< 0.001**	4.69 (0.04)	4.55 (0.04)	4.62 (0.04)	**0.025** **0.015**	4.56 (0.04)	4.50 (0.05)	4.52 (0.05)	0.166 0.178	4.55 (0.05)	4.59 (0.04)	4.50 (0.04)	0.056 **< 0.001**
Whole grains (5)	0.23 (0.03)	0.41 (0.04)	0.44 (0.04)	**< 0.001** **< 0.001**	0.09 (0.02)	0.29 (0.04)	0.29 (0.06)	**< 0.001** **< 0.001**	0.25 (0.04)	0.39 (0.05)	0.41 (0.05)	**< 0.001** **< 0.001**	0.35 (0.05)	0.62 (0.07)	0.68 (0.08)	**< 0.001** **< 0.001**
Milk and dairy (10)	4.67 (0.15)	4.35 (0.14)	4.60 (0.13)	0.901 0.980	4.40 (0.20)	4.43 (0.18)	4.23 (0.18)	0.444 0.405	4.59 (0.18)	4.21 (0.18)	4.43 (0.17)	0.631 0.862	5.48 (0.17)	4.99 (0.18)	5.40 (0.18)	0.745 0.222
Meats, eggs and legumes (10)	8.26 (0.11)	8.54 (0.09)	8.28 (0.09)	0.525 0.903	7.89 (0.15)	8.08 (0.12)	8.29 (0.16)	**0.035** 0.104	8.43 (0.15)	8.69 (0.11)	8.31 (0.13)	0.194 **0.046**	7.93 (0.12)	8.24 (0.15)	8.20 (0.14)	0.495 0.819
Oils (10)^b^	7.15 (0.09)	9.14 (0.08)	9.18 (0.06)	**< 0.001** **< 0.001**	6.75 (0.15)	8.80 (0.13)	9.24 (0.09)	**< 0.001** **< 0.001**	7.27 (0.11)	9.20 (0.11)	9.21 (0.10)	**< 0.001** **< 0.001**	7.14 (0.15)	9.21 (0.11)	9.04 (0.14)	**< 0.001** **< 0.001**
Saturated fat (10)	6.57 (0.13)	6.62 (0.14)	6.59 (0.12)	0.957 0.510	6.60 (0.17)	6.34 (0.15)	6.50 (0.17)	0.577 0.781	6.47 (0.17)	6.63 (0.19)	6.57 (0.17)	0.552 0.172	7.08 (0.15)	6.88 (0.17)	6.74 (0.17)	**0.044** **0.034**
Sodium (10)	2.39 (0.09)	2.60 (0.10)	3.14 (0.10)	**< 0.001** **< 0.001**	2.88 (0.12)	3.38 (0.16)	3.43 (0.15)	**< 0.001** **< 0.001**	2.32 (0.11)	2.49 (0.14)	3.24 (0.15)	**< 0.001** **< 0.001**	2.05 (0.11)	2.30 (0.13)	2.62 (0.15)	**0.001** **< 0.001**
SoFAAS (20)^c^	9.86 (0.31)	8.61 (0.26)	9.48 (0.20)	0.105 **< 0.001**	7.92 (0.39)	5.44 (0.26)	7.62 (0.29)	0.638 0.243	9.84 (0.35)	8.64 (0.36)	9.33 (0.29)	0.151 0.189	12.87 (0.27)	11.93 (0.28)	11.68 (0.27)	**< 0.001** **< 0.001**
BHEI-R (100)	54.70 (0.51)	56.42 (0.50)	58.38 (0.39)	**< 0.001** **< 0.001**	50.76 (0.57)	50.33 (0.42)	54.46 (0.56)	**< 0.001** **< 0.001**	54.64 (0.62)	56.47 (0.66)	58.02 (0.60)	**< 0.001** **< 0.001**	60.79 (0.55)	62.78 (0.57)	63.24 (0.49)	**0.001** **< 0.001**

*Analyzes considered complex sample design for representativeness of the population of São Paulo

**Kruskal-Wallis and nptrend tests. *p* < 0.05

^a^DGOV&L = Dark-green and orange vegetables and legumes

^b^Oils = Vegetable oils. Seed oils and oily fish

^c^SoFAAS = Total calories from solid fat. Alcohol and added sugar

SE = Standard Error

Increases in diet quality scores were observed for the following components: total fruits, whole fruits, whole grains, oils and sodium; there was a reduction followed by an increase in the score of total grain and calories from solid fat, alcohol and added sugar (SoFAAS).

Adolescents presented an increase in the score for the whole grains component between 2003 and 2008, maintaining in 2015. Adults showed an increase between 2003 and 2008, followed by reduction in 2015 in the scores for DGOV&L and meats, eggs and legumes. Older adults had a reduction in the saturated fat and SoFAAS scores. The scores for total fruits, whole fruits, whole grains and sodium showed increases for all age groups over the years. The other components did not present significant differences (Table 2).

Considering that the concentration index of the diet quality in São Paulo population was negative in 2003, we identified better diet quality among individuals of lower income; however, the situation was reversed in 2008 and 2015, resulting in a positive concentration index; representing better diet quality among individuals with higher income in the most recent periods (Table 3). In sum, socioeconomic inequalities determining diet quality initially benefited individuals with lower income in 2003 and, in recent years, individuals with higher income.

**Table 3:** Coefficients of multiple linear regression and global concentration index (CI), according to period of the survey. ISA 2003, 2008, 2015 (SP), Brazil, 2003–2015

Individual characteristics and external factors*	2003		2008		2015	
	β	p**	β	p**	β	p**
Age (Ref. 12–19 years)						
20–59	3.311	< 0.001	6.315	< 0.001	2.379	0.012
≥ 60	7.370	< 0.001	9.522	< 0.001	6.316	< 0.001
Sex (Ref. Male)			0.272	0.700		
Chronic disease (Ref. Absence)	−2.797	0.003				
Physical activity (min/day)	−0.004	0.026			−0.004	0.045
Smoking habit (Ref. Non-smoker)						
Current smoker	−2.285	0.015	−3.261	< 0.001	− 2.335	0.031
Alcohol consumption (Ref. No)					−2.820	0.001
BHEI-R Component - Whole grains	2.078	0.001	2.330	< 0.001	2.413	< 0.001
BHEI-R Component - Sodium			−0.942	< 0.001	−1.000	< 0.001
Health insurance (Ref. Yes)			−1.600	0.048		
Education (years)	−0.254	0.016				
Ethnic group (Ref. Yellow)						
White	−4.772	0.002				
Black	−7.026	< 0.001				
Grayish-brown	−5.196	0.002				
Indigenous	−5.016	0.021				
Household income per capita (in MW) (Ref. < 1 MW)			1.810	0.019	2.039	0.004
Type of residence (Ref. Not living at home)			−2.672	0.012		
Occupation (Ref. Unemployed)			−1.914	0.034		
Marital status (Ref. With partner)					−2.617	0.006
**R** ^**2**^	0.099	< 0.001	0.211	< 0.001	0.200	< 0.001
**CI**	−0.009		0.001		0.005	

*Analyzes considered complex sample design for representativeness of the population of São Paulo

**Multiple linear regression, descriptive level *p* < 0,05

**Fig. 2:**
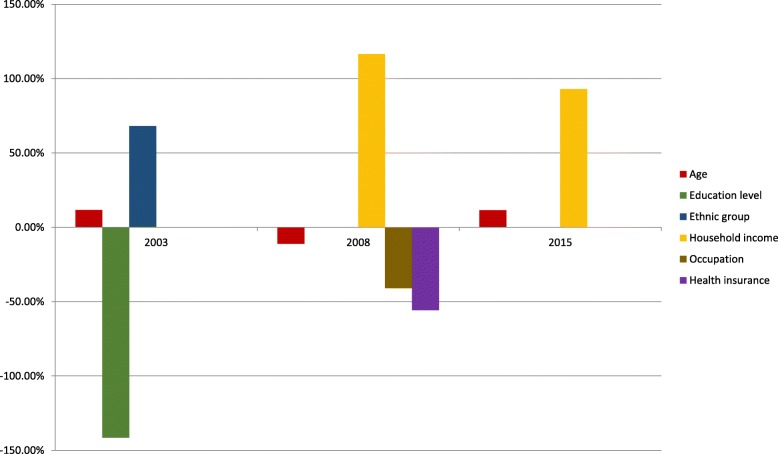
Contribution of main determinants to diet inequality, measured using Concentration Index in Brazilian Health Eating Index. ISA 2003, 2008, 2015 (SP). Brazil. 2003–2015

The results from the determinants of inequalities in diet quality presented statistically significant variables in linear regression models (Table 3). Factors presented positive of the main axis contribute to increasing inequalities in diet quality in favor of individuals with higher income, while negative factors of the main axis contribute to reducing inequalities in diet quality in favor of individuals with lower income (Fig. 2). The main contributor to inequality in diet quality in 2003 was ethnic group; in 2008 and 2015 it was per capita household income (in minimum wages).

## Discussion

The BHEI-R showed a gradual improvement in the period of 12-years among individuals living in the city of São Paulo, and older adults were the age group with the best scores. The main contributors to inequality in diet quality were ethnic group, per capita household income and age. Concentration indices showed that individuals with lower income had higher BHEI-R scores in 2003; however, there was a change in favor of individuals with higher income during 2008 and 2015.

Age is an important factor in determining persistent socioeconomic inequalities in diet quality throughout the surveys analyzed. Although age contributed to increasing inequalities in 2003 and 2015, especially among the poorest, it became a factor contributing to reducing inequalities in 2008.

Adolescents presented poorer diet quality in comparison to other age groups, while older adults had better diet quality over the years. There are several contributing factors for adolescents to remain in this poor nutritional condition [23, 24]. Scientific evidences have shown that adolescents, including Brazilians, with poor diet quality had lower consumption of fiber food sources; increased intake of carbohydrates, fats and foods rich in sugar, salt and fat and high-calorie beverages; lower frequency of breakfast consumption and lower level of physical activity when compared to adolescents with higher diet quality [24–26]. Differences in diet quality between age groups were also identified in the Health and Nutrition Examination Survey 2003–2004 (NHANES), which used the Healthy Eating Index 2005 (HEI-2005); in NHANES, older adults presented better diet quality than young and middle-aged adults [27]. In Brazil, a study conducted among residents of Campinas (São Paulo state) using BHEI-R indicated a progressive increase in the diet quality score simultaneously to the increase in the age of participants [28].

Improvements in diet quality throughout the period analyzed were identified in different age groups, except among adolescents, who had a small reduction in diet quality in 2008. Despite the increase in consumption of total fruit, whole fruit, whole grain and sodium for adolescents, scores obtained are still lower than those identified among adults and older adults. A study evaluating the adherence to a healthy lifestyle among residents of São Paulo city indicated that adolescents have a worse lifestyle, mainly in relation to noncompliance in relation to nutritional recommendations, as measured by BHEI-R [29].

Older adults had higher BHEI-R scores, especially with respect to the higher consumption of total fruits, whole fruits and whole grains. In a cross-sectional population-based study conducted among older adults from Campinas city, a similar trend was observed [30]. Despite having better scores when compared with adolescents and adults, older adults presented reduction in diet quality along the period regarding scores of saturated fat and SoFAAS.

In despite of the general trends in improvement of general diet quality during the period analyzed in the present study, there is still need for changes in the diets of the individuals living in São Paulo city, considering that scores are still below those corresponding to optimal diet quality. Similarly, studies conducted using data from NHANES (1999 to 2000 and 2009 to 2010) and information from population in 187 countries (between 1990 and 2010) suggest that improvements in diet quality identified over time in diverse countries were still insufficient to establish healthy diets [31, 32].

The objective of including diet quality components in the regressions analyses was to highlight the role of socioeconomic inequalities related to dietary quality derived from certain items in usual food consumption; nevertheless, the only items which presented statistical significance was sodium and whole grains; therefore, the other items were excluded from the regressions.

Sodium intake decreased along the period in all age groups; however, the score needs further improvements to reach ideal sodium intake. Changes in sodium intake have also been observed in a previous study among residents of São Paulo city, comparing data from 2003 and 2008 [33], and the present study shows that this trend continued in 2015. In contrast to the data observed for the population of São Paulo, a study on diet quality trends among Americans (1999–2012) observed reduction in the score referring to sodium intake, indicating an increase in diet sodium consumption in the United States [34]. Nevertheless, a study conducted in 187 countries, including Brazil (1990 to 2010), identified that the level of sodium consumed by Brazilians was higher than other Latin American countries, United States, and Canada, exceeding nutritional recommendations [35].

Increased scores were also identified for the components fruits and whole grains throughout the population over the analyzed period. The WHO recommends increasing consumption of these types of foods, due to its contribution to a good diet quality and its association with lower body weight gain; being the ideal consumption of fruits and vegetables 400 g/day [36]. The average consumption of these foods increased from 215.4 g/day in 2003 to 273 g/day in 2015.

Regarding the whole grains component, it contributed significantly to the generation of inequalities in diet quality, since older individuals usually consume higher amounts of whole grains and have higher socioeconomic status in comparison to other age groups in the present study [37].

Income was the main determinant of inequalities from 2008 onwards, replacing the effect of ethnic group in 2003, which indicates changes in the importance of household income during recent years in Brazil. One possible explanation for this finding is the economic crisis occurring in the period, which resulted in higher unemployment rates and reduced household income between individuals from various demographic groups, increasing the impact of income on household consumption decisions. Considering that the economy of São Paulo is predominantly based on commercial and financial activities, the Brazilian economic crisis between 2003 and 2008 resulted in significant losses regarding employment and purchasing power of the citizens.

Since the mid-2000s, Brazil has undergone an important economic slowdown [38]. Such a setback in the economy has impacted household income. According to studies based on data of government revenues derived from income tax from the Brazilian Ministry of Finances during the period from 2006 until 2012, complemented by data from the National Household Sample Survey (NHSS) from 2001 until 2015, income inequalities in Brazil did not display significant reductions over the last decades, despite fluctuations in national income and employment levels; however, recent increases in unemployment rates have affected household consumption decisions [39, 40].

In Latin America and Caribbean countries, ethnic minority groups such as afro-descendant (black and grayish-brown ethnicities) and indigenous groups, still show important social differences and disadvantages compared to white population, observed in lower levels of education, lower average income, higher poverty rates and discrimination in the labor market. They are directly linked to health inequalities, which reflects poorer health conditions for these groups [41]. These ethnic minority groups also present dietary disparities and consequently differences in dietary intake and dietary patterns, resulting in poorer diet quality and lower health outcomes in relation to whites (diets highest in fat and salt and lower in fruits, vegetables and whole grains) [42]. However, disparities are often defined based on ethnicity, factors contributing to disparities may be more associated with socioeconomic level [42].

The relationship between socioeconomic level and ethnicity in the context of diet quality was demonstrated in a study that indicated a higher proportion of individuals with higher diet quality in high socioeconomic level groups and white [43]. In addition, it was observed that factors interfering with food choice, such as convenience, safety, nutrition, and price, are more frequently mentioned as barriers to healthy food consumption between African Americans, when compared to whites [44]. In the present study, individuals from black, grayish-brown and indigene ethnicities had higher impact from socioeconomic inequalities on diet quality in comparison to white individuals, and the yellow ethnicity had no statistical significance in the estimated models.

The evolution of the diet quality of São Paulo city residents showed improvements throughout the 12-years investigated in the present study, and inequalities identified among individuals with different income levels showed that poorer individuals had better diet quality in 2003. However, there was a change in diet quality in favor of individuals with higher income in 2008, and the scenario was maintained in 2015.

Income has been identified as one of the most important factors related to food insecurity [45]. However, while some studies suggest that there is a social gradient in diet quality in favor of individuals with higher income or higher level of education [46–50], others point out that higher income does not necessarily contribute to healthy food consumption and consequent better diet quality [51, 52]. Divergences in the literature are attributable to differences in the units of comparison [53]: healthier diets with lower caloric value present higher costs per calorie, but the diet cost is not higher if portions or meal sizes are considered as measurement units [53].

The present study has some limitations. The BHEI-R calculation was based on the food consumption based on one 24HR, which does not reflect habitual food consumption, or variations in food consumption day-by-day. However, the National Cancer Institute indicates that the use of one 24HR measure is appropriate in the context of studies directed to estimate the usual dietary intake of population groups or differences in usual dietary intake between two or more population groups, considering different days in the week and seasons, as done in the present study [54]. Another limitation was the use of reported household income, due to potential underreporting, especially among the richest individuals. This underreporting may result in higher levels of inequality in income distribution in comparison to actual income distribution [22]. Despite this, referred household income is widely used in national and international surveys, including studies using the income-related inequality approach [55, 56].

This study was designed to comprise complementary data analysis of information collected during ISA 2003 and ISA 2008, including data from the 2015 Health Survey of São Paulo city, i.e., identifying trends in diet quality of urban population in Brazil. The investigation results are based on population-level survey with representative sample of residents of São Paulo city, the largest Brazilian city (corresponding to approximately 10% of the national population), which guarantees internal validity of the study and minimizes selection bias. The methodology applied in the three surveys, as well as the use of sample design and weights, allows comparisons among different periods. Additionally, it is important to point out that the analysis performed in the present study is a useful tool for the investigation of individual characteristics and external factors generating socioeconomic inequalities that influence diet quality from individuals in different socioeconomic groups in any population [22].

## Conclusions

The present study identified improvements in the diet quality of individuals living in São Paulo city between 2003 and 2015, especially among older adults; however, the BHEI-R scores still do not reach the nutritional recommendations. We highlight the need for public policies and interventions in food and nutrition directed to younger individuals, given the poor quality of food consumption among adolescents in the sample. In addition, it is important to notice that changes in the patterns of determination of inequalities according to age, ethnic group or per capita household income during the period analyzed showed the existence of ongoing process of contribution of demographic and socioeconomic factors in the diet quality of individuals living in a large urban center over the years. The social gradient initially identified in diet quality, favoring lower income individuals in 2003, was diluted over time as result of the economic crisis in the country, gradually favoring individuals with higher income and establishing a reverse social determination of diet quality among the population living in São Paulo city.

## Abbreviations

24HR: 24-h dietary recall
BHEI-R: Brazilian Healthy Eating Index
CI: Concentration Index
HEI: Healthy Eating Index
ISA-Capital: Health Survey of São Paulo
MPM: Multiple Pass Method
NDS-R: Nutrition Data System for Research
NHANES: Health and Nutrition Examination Survey
NHSS: National Household Sample Survey
SoFAAS: Calories from solid fat, alcohol and added sugar
WHO: World Health Organization
